# Toxicology: a discipline in need of academic anchoring—the point of view of the German Society of Toxicology

**DOI:** 10.1007/s00204-015-1577-7

**Published:** 2015-08-28

**Authors:** U. Gundert-Remy, H. Barth, A. Bürkle, G. H. Degen, R. Landsiedel

**Affiliations:** Institut für Klinische Pharmakologie und Toxikologie, Charité, Berlin, Germany; Institut für Pharmakologie und Toxikologie, Universität Ulm, Ulm, Germany; Molecular Toxicology Group, Universität Konstanz, Konstanz, Germany; Leibniz-Institut für Arbeitsforschung, TU Dortmund, Dortmund, Germany; Experimentelle Toxikologie und Ökologie BASF SE, Ludwigshafen, Germany

**Keywords:** Toxicology, Translational science, Academia, In silico toxicology, In vitro toxicology, Omics, Regulatory toxicology

## Abstract

The paper describes the importance of toxicology as a discipline, its past achievements, current scientific challenges, and future development. Toxicological expertise is instrumental in the reduction of human health risks arising from chemicals and drugs. Toxicological assessment is needed to evaluate evidence and arguments, whether or not there is a scientific base for concern. The immense success already achieved by toxicological work is exemplified by reduced pollution of air, soil, water, and safer working places. Predominantly predictive toxicological testing is derived from the findings to assess risks to humans and the environment. Assessment of the adversity of molecular effects (including epigenetic effects), the effects of mixtures, and integration of exposure and biokinetics into in vitro testing are emerging challenges for toxicology. Toxicology is a translational science with its base in fundamental science. Academic institutions play an essential part by providing scientific innovation and education of young scientists.

## Introduction

Toxicology was once the science of poisons and intoxication. The scope of topics it covers has broadened and developed with time, however, so that the present-day field of toxicology should be regarded as the ‘science of safety’ (Collins et al. 2008). It is anticipated that the role of toxicology will grow in the future, with contributions to safety assessments of global importance, such as global planning for earth resources, anthropogenic changes in the atmosphere, and modern techniques for food production (Manciocco et al. 2014; Paterson and Lima 2010; UN 2015). For sustainable products, safety assessments must be integrated into product development and the application of new technologies and toxicological evaluations form a key part of this safety evaluation (Sianipar et al. 2013). In summary, toxicology is a translational science, transferring knowledge from basic science into practical applications to safeguard human health and the environment.

The demands on toxicologists are high: along with excellence in their field of basic science—be it biology, chemistry or medicine—they must be capable of providing an integrated view. Translational approaches require a network system in which different specialists work together with a common understanding of the problems and how to solve them.

Today, the pharmaceutical and chemical industries need universities, first, to train toxicologists, and second, to perform research in co-operation with scientists from industry to promote the future development of safety science. New approaches for safety assessments are required, in particular those aiming to reduce animal experiments, with the ultimate vision of developing methods that allow safety assessments without animal testing (Adler et al. 2011; Jennings et al. 2014; Knudsen et al. 2015). Here, academia has to take the scientific lead by building up research networks with industry that should also include scientists from regulatory institutions.

When assessing risks, toxicologists have to critically evaluate findings, drawing on their detailed knowledge of a wide range of methods, experimental skills, and subjects taught at universities. Hence, universities and postgraduate teaching institutions need qualified academic personnel and appropriate laboratory facilities for training and research.

Academia-based research is clearly needed to resolve open questions of great importance. An example is the controversy over the dose–response relationships for endocrine-active substances and the underlying molecular mechanisms. Some scientists believe that the use of all endocrine-active substances should be subject to regulatory control as they claim they have no dose level without an effect. Others, however, feel that endocrine-active substances are also subject to thresholded mechanisms (Testai et al. 2013; Zoeller et al. 2014; Autrup et al. 2015). This issue will remain controversial in the absence of new insights into molecular mechanisms thanks to basic research and how these ‘translate’ into apical endpoints and adverse effects. This is therefore a question of public concern, and research should be performed in independent institutions supported by public money. This example once again highlights the translational nature of toxicology.

Below we describe the different areas in which applied toxicology is in demand. We discuss current scientific questions of relevance that require innovative answers. We also address current topics and challenges in risk assessment and how to handle them. Altogether this should illustrate the overarching nature of toxicology and its role in safe guarding human health and an intact environment.

## Toxicology as an applied science

Toxicology enables the assessment of risks originating from the exposure of humans, animals and the environment to industrial chemicals, plant protection products, biocides, medical drugs and devices, and consumer goods, including food and cosmetics. Recommendations for the protection of workers, consumers, and the environment are derived from scientific investigations and assessments of the toxic properties of these substances.

### Occupational health and safety

Workers must be protected against adverse health effects from hazardous substances at the workplace (Jansen 2003; Kang 2015; Kogi 2015). Toxicologists assess the health hazards of substances at work and combine this with exposure assessments to characterize the risk of adverse health effects. They propose occupational exposure limits to reduce risks to acceptable levels (Ziegler-Skylakakis 2003). Such hazard and risk assessments have to be continuously adapted to new toxicological findings; conventionally, these are observed toxic effects which are progressively supplemented with toxicokinetic and mechanistic data to allow for a more precise assessment of the extent and relevance of toxic effects on humans. Estimates of the recent and future burden of occupational diseases indicate that occupational cancer is still a major problem and will remain so in the future as a result of exposure of workers to carcinogens (European Agency for Safety and Health at Work 2014). Authoritative occupational exposure limits are derived from comprehensive assessments of toxicodynamics, toxicokinetics, mechanistic data, and the evaluation of the underlying experimental data (MAK 2015). At the same time, new experimental investigations are required to complement the toxicological data, incorporating progress made in the toxicological and life sciences (Iavicoli and Boccuni 2010). Toxicologists highly trained in both, the scientific basis and the toxicological methodology, are indispensable for executing these tasks.

### Consumer protection

Consumers are exposed to xenobiotic substances via food, cosmetics and other consumer products such as textiles and toys. Hazardous substances may be present in food as natural constituents or contaminants—of microbial origin or from the environment or food packaging (Wölfle and Pfaff 2010)—residues of pesticides and veterinary drugs, or additives (EFSA 2015a, b; Heberer et al. 2007). The toxicological assessment of xenobiotic substances in food sometimes gains a high level of public interest and presents new scientific challenges, as exemplified by a decade of discussion on bisphenol A (Hengstler et al. 2011; EFSA 2015a, b). Likewise, substances migrating from textiles and toys raise public awareness (SCCS 2009; SCHER 2012). This is especially true if a vulnerable group of the population, e.g. children, may be affected by substances interfering with the development of children or unborn life. Profound toxicological knowledge and a high level of expertise are required to adequately assess the wealth of data on these topics and to draw the right conclusions in order to protect consumers and address public concerns without fuelling fears.

### Environmental health (outdoor and indoor air, drinking water, watercourse, soil)

Breathing air, drinking water, and eating food are major sources of exposure to xenobiotic substances. Limit or guideline values for many environmental substances have been established to prevent adverse human health effects (WHO 2010, 2011). The derivation of these values has to consider potential adverse health effects, but also geogenic contaminants and anthropogenic load. The quality of drinking water and outdoor air in Europe is tightly regulated and regularly monitored; the toxicological risks arising from substances which may be present in water and outdoor air are generally well characterized. Toxicological assessments and recommendations for guideline levels are available for indoor air (SCHER 2007). The toxicological assessment of risks arising from fine dust (respirable particulate matter, PM10) still represents a challenge (Andersen et al. 2012; Raaschou-Nielsen et al. 2011; Shah et al. 2015), and health-based limit values are often exceeded. New challenges arise from rare emissions occurring in accidents and from indirect effects resulting from global warming, e.g. increasing levels of mycotoxins as food contaminants (Paterson and Lima 2010). A comprehensive assessment of environment-related adverse health effects demands profound toxicological expertise combined with a detailed knowledge of environmental emissions and immissions.

### Safety pharmacology

Drug toxicology (also termed pharmaceutical toxicology or safety pharmacology) is the study of the potential undesirable effects of drug candidates and drugs in the therapeutic range and above. This is a prerequisite for clinical studies. Traditionally, toxicological testing of new drugs was performed according to a standard study programme (Kinter and Valentin 2002; Pugsley 2005). More recently, pre-clinical testing has been tailored to the specific properties and mode-of-action of the new drug and may be adapted based on the outcome of previous studies (Pugsley et al. 2011). This flexible, tailor-made approach is accommodated by the regulatory registration process. New types of drugs (e.g. oligonucleotide therapeutics and stem cells) present new challenges and demand newly developed test systems and novel concepts to assess the results of these studies (Muller and Milton 2012).

In drug development projects, toxicology—closely linked with pharmacology and pharmacokinetics—is decisive in the success (or failure) of a new drug candidate during pre-clinical and early clinical testing. Any misinterpretation of substance properties cannot be rectified in the later stages and thus causes expensive failure of the whole project. Toxicology therefore generates value by timely elimination of non-eligible drug candidates (‘fail early and cheap’) and enables reliable risk assessments and risk management in early clinical trials. The notion that toxicology ‘kills’ drug candidates is obsolete; what toxicology actually does is to push drug development projects towards valuable drug candidates.

### Clinical toxicology

Clinical toxicology links preventive and experimental toxicology with clinical medicine. This area of toxicology has undergone profound changes during the last decades: traditionally clinical toxicology was concerned with the diagnosis and treatment of acute intoxications (i.e. reduction of absorption, accelerated elimination, and antidotes). Hence, clinical toxicology was used primarily by emergency physicians (Hahn 2009). The incidence of acute intoxications has decreased, however, during the past few decades due to the development of safer products (a merit of toxicological research), especially safer drugs with a wide therapeutic index. However, effective antidotes for acute intoxications, e.g. for death cap fungus (*Amanita phalloides*) poisoning, are still needed. This requires a detailed knowledge of fungal toxins (amongst others, α-amanitin) and their toxic mechanisms, as well as repair mechanisms in the damaged organ (the liver) (Müller and Desel 2013). The investigation of further toxins of animal, plant and microbial origin (e.g. botulism) and infections (e.g. *Clostridium difficile*-associated diarrhoea and diphtheria) has revealed their toxic mechanisms and paved the way for future potential therapies and applications in drug design, e.g. ‘molecular Trojan horses’ (Barth and Stiles 2008).

Competence in clinical toxicology comes together in poison control centres which gather experimental and clinical data on a wide range of different substances (Sutter et al. 2010). Poison control centres provide toxicological risk assessments after acute and chronic exposure covering cases with mild intoxication or subclinical intoxication. Rapid and reliable information on the toxicological profile of a substance is the basis for recommendations of effective treatments (or giving the ‘all-clear’) and has significantly improved medical care.

## Current scientific topics in toxicology

### Toxicological research in the past

In the past, toxicological research was not mainly preventive. The need for this did not become evident until exposure-related severe health impairments had been observed in many people. Examples are the well-known disasters due to thalidomide (Contergan^®^, induced phocomelia and amelia), asbestos (lung cancer and pleural mesothelioma), aromatic amines (cancer of the urinary bladder in painters and industry workers), arsenic (skin cancer), and contact allergies to substances encountered in everyday life, and in the environment. Research into asbestos, which was started after recognizing asbestos-induced cancer, resulted in new knowledge on the mode of action not only of asbestos fibres, but of fibres in general.

It was demonstrated that bio-persistent fibres with a certain geometry—‘critical fibres’—induce chronic inflammation in the lungs due to their ability to trigger incomplete (‘frustrated’) phagocytosis associated with massive activation of macrophages and release of pro-inflammatory mediators (Gibbs and Hwang 1980; Rom et al. 1991). This knowledge led to the recognition that newly developed nanomaterials with an asbestos-like geometry might cause similar effects. It was therefore possible to prevent the induction of lung damage by such materials during their development and a long time before they became commercially available (Gebel et al. 2014).

It became evident that the severe health impairment and disease in humans exposed to the above-mentioned substances occurred because there was not enough knowledge on the toxicological profile of these substances to enable the risks to be recognized, which would have resulted in concrete advice on minimization of exposure.

A principal aim of toxicological research, therefore, is to identify potential risk factors before exposure of humans to toxic substances, in particular where the damage or disease occurs after a long latency period. A paradigm change is therefore needed here to establish the concept of preventive toxicology.

### Selected areas of current toxicological research

#### Carcinogenesis: mechanisms and threshold values

In real life, humans are exposed to a variety of carcinogenic substances in food, in the environment, and at the workplace. According to the current paradigm for genotoxic carcinogens, it would be best to completely avoid exposure to such substances. There are, however, mechanism-based arguments that complete prevention of exposure might not be necessary (Johnson et al. 2009). This means that identification of the most critical carcinogens, elucidation of the molecular mechanism underlying their adverse mode of action, and of cellular repair mechanisms are extremely important (Oberemm et al. 2009). The knowledge of such mechanisms enables the decision whether threshold levels can be identified below which no carcinogenic effects should be expected (Bolt et al. 2004). The identification of the molecular and cellular mechanisms underlying the cancer-inducing potential of chemicals is the crucial step in science-based risk management.

#### Endocrine-active compounds and non-monotonic dose responses

Endocrine-active substances can interfere with the endocrine system by mimicking hormones or inhibiting their functions, or by stimulating or inhibiting the synthesis of hormones in the body. The relevance of endocrine-active substances for human health is a subject of controversy (Lamb and Boffetta 2014). Many scientists believe that thresholds exist and that normal risk assessment paradigms are appropriate for these substances, also in the light of very low exposure levels and the low intrinsic activity of most endocrine-active substances (Autrup et al. 2015; Borgert et al. 2013; Testai et al. 2013). These authors emphasize that the extensive database for the potent hormone diethylstilbestrol is consistent with dose-related effects for adverse outcomes in animals and humans. Other authors do see a risk because of the combined effects of various similarly acting substances and suggest a non-monotonic dose–response model for hormonally active compounds, implying that low-level exposure conditions might be relevant (Vandenberg et al. 2012; Zoeller et al. 2014). Recently, a possible contribution of endocrine-active compounds to the increasing prevalence of diabetes and obesity has been discussed.

The concerns about potential endocrine-mediated health hazards have resulted in many activities in the field of regulatory toxicology (e.g. EFSA 2013; SCCS 2015). The Organization for Economic Cooperation and Development (OECD) in their ‘Conceptual Framework for Testing and Assessment of Endocrine Disruptors’ gives information on data sources and the different test systems available and under development. The OECD has developed, optimized, and validated numerous in vitro and in vivo methods for testing the endocrine activity of substances and related effects (mammalian and non-mammalian toxicity). These activities must be continued in order to develop novel assays based on a better understanding of the mechanisms underlying the regulation of endocrine effects in the organism. The current controversies demonstrate the urgent need for further basic research to elucidate the discrepancies between the results obtained by in vitro test systems and animal studies, and their relevance for humans. Most important in this context is a better understanding of hormonal signalling pathways and related disorders, in particular those for the thyroid hormones, and the detection of such disorders by appropriate test systems (EFSA 2013).

#### Toxicity of mixtures of substances

Toxicological research has so far focused on investigating the mechanisms by which single compounds interfere with biological processes. However, in real life, humans are exposed to a variety of compounds at the same time, for example at the workplace, or via (multiple) contaminants in food, in the air, or the environment. Several approaches have been proposed to take into account the action of combined substances.

Previous (non-systematic) studies suggested that the adverse effects of mixtures of toxins were smaller than would be expected from addition of the individual effects caused by the single substances in mixtures (‘additive model’) (Hertzberg et al. 2013). However, there are well-known exceptions: substances inducing enzymes, which metabolize and thereby activate chemicals, show stronger than additive effects in combination with their substrate chemicals; the same holds true for combinations of genotoxic carcinogens in combination with substances that inhibit DNA repair systems (Kortenkamp et al. 2012; Ermler et al. 2014).

Some of the approaches suggested are rather pragmatically oriented and lack a scientific basis for the toxicological assessment of the action of a combination of chemicals. Combined effects caused by mixtures of substances are especially relevant in the combined assessment of human health, and further research is needed to establish a knowledge-based system of assessing potential adverse effects caused by simultaneous exposure to different toxic substances at levels below their individual NOAEL (SCCS, SCHER, SCENIHR 2011). The substance mixtures requiring investigation could be ranked by urgency based on our knowledge of human exposure to such mixtures in food and drugs, at the workplace, and in the environment (Carlin et al. 2013).

#### Immunotoxicity

Adverse effects of substances can be mediated by the immune system (IPCS 2012; Rooney et al. 2012). A reaction of the immune system contributes to conditions such as allergic contact dermatitis and asthmatic symptoms. With a prevalence of about 15 %, contact dermatitis is amongst the most frequent diseases induced by chemicals, including cosmetic ingredients (Belloni-Fortina et al. 2015). Sensitization testing is essential to be able to warn and to prevent exposure. Because animal testing for cosmetic products and their ingredients has been banned since 2013 (EU 2003), much has been invested in the development of novel toxicological test systems to address this endpoint (Mehling et al. 2012; Basketter et al. 2013; Reuter et al. 2015).

Besides sensitization reactions, the immune system is also involved in ‘idiosyncratic’ toxicity of the liver (Chalasani and Björnsson 2010). The immune system is trained to recognize and eliminate foreign structures. Genome-wide comparison with our phylogenetically closest relatives suggests a significant evolutionary pressure to develop a highly efficient immune system. This comes, however, at a cost, i.e. frequent and undesirable immunoreactions following exposure of humans to chemicals. To be able to predict such immunotoxic reactions, significant future efforts in the field of basic research are required. One complication, however, is that the transfer of results from animal models, in particular mouse models, to the human situation is rarely possible. It recently became evident that inflammatory processes in the brain play a role in acute events in this organ (ischaemia), but also in age-related neurodegenerative diseases such as Alzheimer’s, and there are hints that the reaction of the brain tissue towards substances encountered in daily life might play a role in these processes (Heneka et al. 2015). Because of the dramatic increase in neurodegenerative diseases, this topic will become an important interdisciplinary field of activity requiring toxicological expertise.

#### Toxicity of nanomaterials (nanotoxicology)

Nanotechnology is one of the key technologies of the twenty-first century. Ongoing toxicological research on nanomaterials has resulted in sustainable development of such materials and their broad acceptance by consumers. Nanomaterials are not only used as industrial intermediates, but also used for consumer-oriented everyday products (such as cosmetics, food, packaging, clothing). Moreover, nanoparticles are considered useful tools in the field of medical therapy and diagnostics, e.g. ferric oxide nanoparticles as transporters for anticancer drugs or as contrast agents for magnetic resonance imaging (Abakumov et al. 2015).

Toxicological investigations to assess the risk of newly developed nanoparticle systems are challenging tasks at the interface between biology, medicine, pharmacy, chemistry, and physics. Existing test guidelines for chemicals are generally applicable to nanomaterials, but may need amendment with regard to test item preparation and characterization. Depending on the route of exposure (dermal, oral, inhalation, intravascular for medically used particles), the choice of the relevant test system plays an important role. Surprisingly, in vitro methods play only a minor role in nanotoxicology assessments, most likely because of the limited characterization of nanomaterials in test systems, the choice of non-relevant concentrations of the test material, and insufficient knowledge of the dose in vitro. For the toxicological assessment of nanomaterials, both the variety of materials and their different applications pose challenges, and it will not be possible to completely test every nanomaterial in every test system. Strategies for grouping nanomaterials for toxicological assessment are therefore required (Gebel et al. 2014; Arts et al. 2014, 2015; Godwin et al. 2015). Important in the context of nanotoxicological research are questions regarding the uptake, mobility and deposition of nanomaterials in the body, the effects of nanomaterials after inhalation and oral intake, and the long-term effects of nanomaterials (Savolainen et al. 2013). Currently, a comprehensive research consortium is investigating the long-term effects of nanoparticles after their uptake into the body via inhalation (Gebel et al. 2014).

#### Developmental neurotoxicity

There is evidence that disorders of intellectual abilities and mental health are increasing. A committee of the US National Research Council has estimated that developmental disorders are to 3 % a consequence of an exposure against environmental influences and further 25 % a direct consequence of an interaction between environmental factors and genetic pre-disposition (see Landrigan et al. 2012). Since the existing toxicological routine test systems are not suitable to detect this kind of developmental disorders, there is pressure need to develop more adequate test systems, which are sensitive enough on the one hand (Balmer et al. 2014; Zimmer et al. 2014) and enable the interpretation whether the obtained results are relevant for humans.

Although an OECD guideline (TG 426 *Developmental Neurotoxicity study*) was established more than 10 years ago, there is still significant missing knowledge on this topic, i.e. there is an urgent need to develop adequate methods (Makris et al. 2009; Saunders et al. 2012). It is worth noting that the interpretation of data from animal studies is very difficult, and decisions whether subtle results from animal studies are relevant or not for humans require elaborate, long-term epidemiological studies.

Research into developmental neurotoxicity must be deepened and presents a particular challenge because of the large number of potentially harmful chemicals in widespread use, and because of the controversy on how to interpret results from animal studies (Berghuis et al. 2015).

## New challenges and novel approaches

### In silico toxicology

In silico toxicology, also known as computational toxicology, is a novel strategy in toxicology which aims to establish mathematical models based on existing knowledge and to use these for making predictions. A feature of in silico toxicology is its high degree of interdisciplinarity, linking modern toxicology with bioinformatic and chemoinformatic methodologies. The concept that the biological activity of a chemical compound is implicitly determined by its chemical structure is, as such, not new (Collins et al. 2008). Only a few years ago, the term ‘in silico toxicology’ was mostly used for the prediction of potential toxicity based on physicochemical or structural properties of chemicals. Nowadays, the term is used in a wider sense and covers predictions based purely on structure, as well as toxicity or more general effect profiles.

Databases with information on chemical structure and effect, and chemical structure and adverse modes of action (including data from high-throughput technologies on adverse outcome pathways), form the basis for the recognition of some patterns that may be generalized. In theory, a new rule for an expert system or a quantitative structure–activity relationship can be deduced for each toxicological endpoint via data evaluation and statistical analyses. If, in an ideal situation, the toxic effect can be associated with a known mechanism of action, MOA, or—even better—if links with an ‘adverse outcome pathway’ can be established, this type of statistical analysis is promising. If, however, the toxic effect is the result of several different mechanisms working sequentially or simultaneously, then reliable prediction is still difficult at present (Knudsen et al. 2015).

In principle, one can distinguish between (a) evidence-based expert systems that derive rules concerning the relationship of structure and toxicity from existing data, based on the recognition of ‘structural alerts’; and (b) statistics-based systems that use descriptors (structural and physicochemical parameters) to derive quantitative correlations between structure and effect.

In silico methods require high-performance data storage and computing and have therefore developed only recently. The increasing number of publications in literature databases (e.g. PubMed) over the last 10 years on the topic ‘QSAR’ and ‘in silico’ highlights their increasing importance. Likewise, the volume of funding for this kind of research, e.g. through EU grants, has been high. For instance, in silico methods play a central role in the European SEURAT-1 Research Initiative and the ‘eTOX’ project funded by the Innovative Medicines Initiative. In the context of the reorientation of Toxicology (TOXCAST und ‘tox21’) promoted by the US Environmental Protection Agency (EPA), in silico toxicology is playing a major part in the paradigm change away from animal testing towards toxicological assessment of alternatives by using a combination of molecular biology and ‘omics’ techniques including computational methods.

### In vitro methods

Primary cell cultures have always been the gold standard for in vitro testing. However, conventional two-dimensional (2D) cultures of primary cells tend to undergo de-differentiation and lose their organotypic functions. More complex techniques of cell culturing, including 3D cultures (e.g. collagen sandwich models, co-cultures, hanging drop, spheroids) and tissue engineering are capable of mirroring complex cell–cell interactions (Alépée et al. 2014). The priority is to mirror the natural context of the tissues of an organ. Commercial application of such systems has already started, and these systems display dose–effect profiles that are indeed closer to in vivo systems than classical cell culture methods.

New developments in the area of dynamic cell culture using bioreactors are also enabling the in vivo-like transport of compounds in an in vivo-like tissue context, thus going one step forward towards more realistic exposure scenarios. By miniaturizing such systems, bioreactors are likely to become more broadly applicable. Such ‘microfluidic’ systems are the basis for the development of ‘organ-on-a-chip’ models (Kelm and Marchan 2014). In such assemblies, multichannel 3D microfluidic cell culture chips are used, which comprise all or at least a representative fraction of cell types in a tissue in their natural 3D context. Based on their dynamic culture conditions, such systems are able to mirror the activity status and also the mechanistic and physiological reactions of an organ. As a next step, several organ systems can be combined to enable systemic investigation of toxic effects.

In view of the limited access to human tissue, stem cells are likely to gain more and more importance as a replacement for primary human cells. Much work is still needed to optimize techniques based on embryonic stem cells and induced pluripotent stem cells (iPS) before the properties and activities of such cell models are similar to those of the corresponding primary cells. Cells from animals, representative cell lines, and genetically modified cells have played an important role in the validation of methods and will continue to do so (Adler et al. 2011).

Predictivity for the in vivo situation in humans with modern in vitro methods is steadily growing. Unfortunately, however, in vitro methods that are rather close to in vivo often fail due to technical limitations in assessing their validity (standardization and overall reproducibility). In complex culture systems, specific key aspects of biological processes are gaining more and more attention, but at the same time the standardization, quality control and interpretation of results is becoming more difficult. For this reason, modern in vitro methods should be kept as simple as possible but as sophisticated as necessary, to guarantee satisfactory answers to future scientific questions. Integrated testing strategies (ITS), as opposed to single definitive tests, are expected to efficiently combine different in vitro methods. Validation of modern in vitro methods has been and will continue to be a challenge (Jennings et al. 2014; Rovida et al. 2015).

### ‘Omics’ methods and their interpretation

In scientific research, the term ‘Omics’ analysis refers to the parallel assessment of all molecules or modifications of molecules of a certain type in a given sample. Examples are the assessment of all RNAs (transcriptomics), proteins (proteomics), or metabolites (metabonomics, also called metabolomics). Meanwhile, also epigenetic changes, such as cytosine methylation in DNA or various kinds of posttranslational modifications of histones, can be detected via ‘epigenomics’. Applying such methods in toxicological research enables the comparative analysis of substance-induced changes at various molecular levels in a in a single study.

Apart from the biological model and the technology used for measurement of gene expression, the strategy for statistical analysis and interpretation are of crucial importance in toxicogenomic experiments (Rahnenführer and Leist 2015). Often software is being used that, on the one hand, produces lists of significantly deregulated genes and, on the other, enables more complex analyses such as testing for overrepresentation of certain functions and signalling cascades. How to find the best biological interpretation of the massive amounts of complex datasets and to view these from the toxicological perspective is a challenge, even for the most highly skilled professionals, and clearly calls for the expertise of toxicologists trained in both classic and modern toxicology. Routine deployment of such techniques in drug development is not yet possible.

Existing data may yield information on dose-dependent (in vivo) or concentration-dependent (in vitro) biological responses. The focus is on the elucidation of mechanisms underlying toxic effects, of both individual compounds and classes of compounds (Andersen et al. 2015).

In the area of cellular deregulation, usage of the above methods aims to identify molecular signatures for the classification of adverse effects in vivo and for prediction. But since non-adverse influences, such as eating or a change of culture media, can also lead to purely adaptive responses in ‘omics’ studies—which may come close to the level of deregulation seen after drug treatment—it is inappropriate to interpret any drug-induced change in expression as an *adverse* effect.

An important and challenging task of basic science therefore is to identify the dose (or concentration) range where responses have primarily adaptive functions, depending on the magnitude and duration of the response, and on the simultaneous activation of other signalling pathways. It will certainly not be possible to generally deduce from the absence of changes in the target cells that a given substance is harmless at the chosen dose. If, however, it were possible to establish such a relation for a specific form of toxicity, this could help simplify future toxicological risk assessment (Oberemm et al. 2005; Ellinger-Ziegelbauer et al. 2008; Thomas et al. 2011).

A number of applications highlight the aims that may be pursued with these methods, such as (a) diagnostic classification of organ damage or prediction of chronic changes based on changes in gene expression in experimental studies. One potential use is the prediction of carcinogenicity in the context of prioritizing chemicals for long-term studies, in view of the large number of compounds awaiting testing; (b) establishment of ‘health-based guidance values’ such as reference dose, ADI or TDI, in which, based on deregulated genes or their functions or signalling pathways, a point of departure is determined via ‘benchmark dose modelling’ and used to derive the respective health-based guidance value; (c) prediction and identification of toxicity via metabolite profiling; (d) characterization of ‘toxicological pathways’ (adverse outcome pathways), which are viewed as one of the most important results and tools of the science of toxicology in the twenty-first century and are considered fundamental to a better general classification of the effects of chemicals (Burden et al. 2015).

### Implementation of physiologically based toxicokinetic modelling

Toxicokinetics relies on the same principles as *pharmaco*kinetics, but there are differences in data availability and aims. In *pharmaco*kinetics, the typical approach is to run experimental studies yielding large amounts of data. For *toxico*kinetics, however, this procedure is not suitable. Instead, physiologically based kinetic models have gained special importance for toxicology and have been recognized as a valuable tool to explain toxicity phenomena and underlying mechanisms (Bessems et al. 2014). Physiologically based (toxico-)kinetic modelling (PBTK) necessitates (a) a systemic approach that can mirror physiological processes of kinetics in a structural and a mathematical model; and (b) implementing the numerical algorithms by flexible programing that enables use in various scenarios. Using a ‘bottom-up’ approach, individual compound-specific parameters are determined in independent systems, often in vitro or in silico, and then incorporated into a single model for the whole body. PBTK models have the great advantage that—based on knowledge of physiological processes—the effect of changes in physiology can be predicted on the concentration–time course, even in an individual organ. Changes in physiology may include reduction of xenobiotic metabolism in premature infants or changes in pregnancy. Coupled with dose–effect models, prediction of effects and their magnitude is possible (Mielke and Gundert-Remy 2012). A promising area of future development is the implementation of interindividual variability, which can predict the behaviour of the general population across all age classes and special features (Zeise et al. 2013); another area is to establish new structural models for the prediction of the time course of intracellular drug concentrations as well as in cell organelles that are linked with the mechanisms of action. The same is true for modelling of the time course of concentrations in in vitro systems, also called reverse PBTK or in vivo–in vitro extrapolation (Coecke et al. 2013). The latter approach is based upon the idea that the toxic effect is more closely linked with the time course of drug concentration at the site of action than with external exposure, as determined in food, in the ambient air at the workplace, or in cell culture medium in in vitro systems.

Implementation of this approach and its further development as an interdisciplinary activity is a promising step towards the development of both in vitro systems (Schug et al. 2013) and a new pillar for improved risk assessment. Many of these new approaches have been set up and evaluated in non-academic institutes of toxicology, indicating that university departments are lagging behind in their expertise.

## Current scientific topics in risk assessment

### Impact and significance of alternative methods

In silico methods and in vitro systems are traditionally much used approaches to complement or partly replace animal tests in toxicology. Long established are in vitro genotoxicity tests that allow conclusions to be drawn on the genotoxic and mutagenic properties of a chemical, often without the need for further in vivo testing. Also established are several alternative methods for eye and skin irritation testing and the use of primary hepatocytes for metabolism and interaction studies (Adler et al. 2011; Basketter et al. 2012; JRC 2014).

In view of (a) test requirements for a large number of chemicals in the REACh Regulation, (b) limited capacities for in vivo toxicity testing, and (c) for reasons of animal protection, further development of reliable and time-saving alternative (non-animal) tests is of particular importance, let alone the political interest in animal protection (EU 2003). Compared to in vivo models, the in vitro methods are often reductionist, less complex models, and have limitations when the interplay between multiple cell types (e.g. in liver toxicity) or various tissues (e.g. in endocrine deregulation) causes a toxic effect (Lilienblum et al. 2008).

Often ‘integrated test strategies’ which combine in silico, in vitro, and in vivo methods allow the best predictions of toxicity in humans. Against this background and because of the ban on animal tests for toxicity testing of cosmetic ingredients, several EU-funded programmes have been initiated, one of the aims being to develop in vitro systems for the detection of organ toxicity (Andersen et al. 2015).

Unfortunately, these research programmes are reaching their limits in some cases due to politically motivated restrictions on conducting research exclusively with human cell systems. Accompanying research in animal models was not sponsored by such EU-funded research programmes. The result is that it has been very difficult to assess the in vivo relevance of the data generated for numerous substances with human in vitro systems only. Thus, it is necessary to conduct additional studies in the context of a classical ‘parallelogram strategy’ to assess (a) the in vivo relevance of in vitro rodent systems data; and (b) the human relevance of in vitro rodent systems. Thereby, then several alternative test methods developed within the EU programme can be assessed for their in vivo relevance.

### Exposure: external versus internal exposure

For a toxicological risk characterization, along with considerations of hazardous properties, mode of action and dose–response characteristics of a given compound, exposure assessment is an indispensable element. It must take into account all intake routes (by inhalation, oral, dermal). Depending upon the existing compound concentrations, its physicochemical properties and the intake route, such *external* exposure, will result in different *internal* exposures.

Sensitive analytical methods serve to determine contaminant levels present in various media (e.g. air and food) and allow conclusions on *external* exposure or for the surveillance of limit values for hazardous chemicals at the workplace (OELs). To assess *internal* exposure, two approaches can be taken: (a) physiologically based toxicokinetic modelling (PBTK, see above); and (b) compound-specific analysis in biological samples (blood, urine) collected in human biomonitoring (HBM) studies. Both (complementary) approaches require special expertise. The relevance of PBTK and HBM for scientifically based risk assessment is beyond dispute and will gain increasing international importance (Aylward et al. 2013).

HBM studies assess existing exposure from all sources (*aggregate exposure*) in populations. Since biomarker data reflect individual factors such as intake, metabolism, and excretion, HBM is an improvement over conventional estimates of (external) exposure. HBM studies with a suitable design can also reveal trends over time in contaminant burden or in subpopulations (Den Hond et al. 2015). Newly developed methods with simultaneous analysis of biomarkers for groups of chemicals (e.g. mycotoxins, phthalates, preservatives) will enable better surveys on combined human exposures (see above, mixtures).

The opportunity of improved exposure assessment by HBM also presents new challenges with regard to interpretation and communication of data (Exley et al. 2014). The detection of a chemical or its metabolite in body fluids by highly sensitive analytical methods is not equivalent to a ‘danger’. The careful scientific interpretation of biomonitoring results needs to take into account the data on dose–effect relationships from animal studies, and, to allow for a human equivalence dose approach, the internal concentration related to the doses.

### Sensitive subgroups/individuals in the population

Whether exposure to chemicals presents a health risk in an individual or a population is evaluated on the basis of different factors, i.e. data on adverse effects, the dose–effect relationship, the dose without any adverse effects, and the extent of human exposure. It is obvious that the extent of exposure varies between individuals: newborns and infants have a higher food intake in relation to body weight than adults, which is an important aspect in food contaminant assessments. Infants have a higher ratio of body surface area to body mass index than older children and adults, which may lead to a higher dermal uptake per kg body weight. Moreover, when excretion of xenobiotics is slower, the same external dose can result in higher internal concentrations (internal exposure) than in adults (Abraham et al. 2005; Mielke et al. 2005). Due to these factors, infants and toddlers are regarded as a sensitive subgroup in the population. Whereas means are at hand to correct the safe dose for the differences in kinetics, it is more difficult to take into account the potentially higher sensitivity of the developing organism and possible irreversible effects later in life (WHO 2006). Special attention is therefore paid to chemical exposure during the pre- and postnatal stages (‘critical windows of exposure’), notably in a risk evaluation of endocrine-active compounds. So far, there are no widely accepted and human-relevant test methods for important functions such as immunocompetence and intellectual development. There is as yet no consensus whether persons of advanced age need to be considered separately in risk assessments. With regard to exposure via food, separate exposure values are already determined for ‘the elderly’ (>65 years) and occasionally ‘the very elderly’ (>80 years).

Genetic polymorphisms in xenobiotic-metabolizing enzymes and transport proteins in individuals or subpopulations have been known for quite some time as the toxicokinetic basis for a higher sensitivity to certain drugs or chemicals (Scheuplein et al. 2002). To account for this when evaluating toxic chemicals and deriving ‘safe’ doses and exposure periods, procedures have been set by convention which arrive, however, at overly conservative estimates (Alexeeff and Marty 2008; Dourson et al. 2002; Ginsberg et al. 2004). Improved statistical modelling could lead to more realistic estimates. Also known are genetic polymorphisms in genes, e.g. DNA repair genes, which modulate the effects of xenobiotics (Woo et al. 2014). Further conceptual work is, however, needed to establish how this knowledge can be quantitatively implemented.

### Adaptive (responses) and adverse changes

Toxicological risk assessment is not a rigid procedure or routine; instead it incorporates scientific developments and new conceptual questions as they occur. One important task in the context of novel methods of characterizing effects of chemicals (see section "New challenges and novel approaches") will be to develop tools to define called pathways of toxicity or adverse outcome pathways (Burden et al. 2015). Furthermore, the development of criteria to distinguish between *adaptive* responses and *adverse* changes (Keller et al. 2012) will become extremely important. The background for the latter are changes observed in ‘omics’ studies where harmless influences, e.g. feeding or exchanging culture medium, led to expression changes which may have come close to the level of drug-induced deregulation in cells (see section “‘Omics’ methods and their interpretation”) (Zhang et al. 2014). Valid evaluation in the future will therefore have to be supported by more extensive basic research.

## Conclusions

Toxicology is a translational science and an academic discipline in its own right. As a scientific discipline, toxicology aims to discover the mechanisms underlying health impairments caused by substances. Basic research is essential to this task.

Basic research that investigates and identifies the mechanisms by which exposure to substances interferes with the functions of biomacromolecules, cells, organs, organism and—in a wider sense—ecosystems, and the consequences of such dysfunctions, should be directed towards the final objective, namely how the results can be translated into safeguarding human health. This means that applied toxicological test systems have to demonstrate their robustness, significance and relevance: in vivo relevance must be defined for in vitro *test* systems, and for animal models the relevance of the research results for humans must be established.

Much effort has been invested in establishing the concept of ‘preventive toxicology’ over the past few decades. In doing so, potential risk factors have been identified using the most modern and sensitive methods. This concept should be expanded in such a way that safety assessments are integrated into the development of new technologies. Toxicology will cover the potential effects on human health in this concept.

To meet the challenges of this concept, toxicology needs constant improvements in methodology and implementation of novel approaches, including (a) in silico toxicology (computational toxicology); (b) in vitro methods complementing and replacing animal testing; (c) ‘omics’ approaches including transcriptomics, proteomics, metabonomics, and ‘epigenomics’; and (d) physiologically based (toxico-)kinetic modelling. Implementation of such approaches and their further development and refinement will improve the assessment of risks to human health. Success, however, clearly depends on interdisciplinary collaboration, as these new approaches require a broad array of expertise in different areas of basic scientific research. Many of these approaches are currently being established and evaluated in non-academic institutes of toxicology, which indicates that university departments are lagging behind. Academia should be taking the lead in setting up networks with industry and regulatory institutions in order to fulfil its role in the education of young researchers. Toxicological research at the university level should be strengthened, and the discipline should be supported by the authorities responsible for the environment and the health of consumers and workers. Even the authorities responsible for economic development should be interested in sound toxicological science as a basis for innovative and sustainable products.
